# Unveiling triterpenoid superiority in a newly developed *Ganoderma lucidum* variety through untargeted metabolomics approach

**DOI:** 10.3389/fnut.2025.1541162

**Published:** 2025-04-10

**Authors:** Zhibin Pan, Junhan Lin, Gelian Luo, Weiqing Cheng, Ye Li, Changhui Wu

**Affiliations:** ^1^Fujian Vocational College of Bioengineering, Fuzhou, China; ^2^GanoHerb Co. Ltd., Fuzhou, China

**Keywords:** *Ganoderma lucidum*, fruiting body, triterpenoid profile, UPLC-Q-Orbitrap-MS, PCA

## Abstract

The fruiting bodies of *Ganoderma lucidum* are renowned for their therapeutic properties, primarily due to their triterpenoid content. Variability in *G. lucidum* strains may influence the composition and abundance of triterpenoids. In this study, we explored the triterpenoid superiority in a newly developed *G. lucidum* strain (GL_V2) obtained through mutation breeding, and compared it to a widely cultivated strain (GL_V1). GL_V2 exhibited a 1.4-fold increase in total triterpenoid content and higher DPPH radical scavenging activity compared to GL_V1, while polysaccharide levels remained consistent. Using UPLC-Q-Orbitrap-MS and chemometric analyses, we identified 589 metabolites, including 86 triterpenoids. Multivariate statistical analyses revealed clear differences in overall metabolite profiles and triterpenoid compositions between the two strains. OPLS-DA identified 56 triterpenoids as key distinguishing markers with VIP values above 1.0. Notably, GL_V2 exhibited increased levels of seven ganoderic acids, two ganoderiols, three ganolucidic acids, and two ganosporelactones, while GL_V1 showed higher concentrations of six lucidenic acids. These results highlight the superior triterpenoid composition of GL_V2 and its potential for developing more potent *G. lucidum*-derived products. This study offers valuable insights into varietal differences in triterpenoid profiles and their implications for the cultivation and therapeutic use of *G. lucidum*. Additionally, the findings of this study suggest that GL_V2 holds significant potential for the development of more effective nutraceutical and pharmaceutical products derived from *G. lucidum*.

## 1 Introduction

*Ganoderma lucidum,* a basidiomycete fungus, is widely distributed across temperate and tropical regions in Asia, Europe, and North America. In Asian cultures, including China, Japan, Korea, and Vietnam, *G. lucidum* serves both medicinal and culinary purposes for a long history of time. It has been employed in medicinal practices in China for over two millennia. It was first documented in the earliest classic book of Chinese medicine, “Shen Nong Ben Cao Jing” (1). The fruiting bodies, mycelia, and spores of *G. lucidum* are esteemed as one of the most renowned medicinal fungi globally (2). The commercial valuation of *G. lucidum* is estimated to exceed 2.5 billion U.S. dollars (3). Scientific research has verified its traditional applications, attributing its effects to diverse bioactive constituents such as triterpenes, polysaccharides, steroids, phenolics, alkaloids, fatty acids, and amino acids (4–7). These compounds exhibit a wide range of pharmacological benefits, such as anti-tumor (8), immunomodulatory (9), anti-oxidant (10), anti-bacterial (11), and anti-inflammatory (12) properties.

Among various bioactive compounds in *G. lucidum*, triterpenoids have emerged as a central focus of modern scientific research (4, 13, 14). Triterpenoids are a diverse class of organic compounds derived from the isoprene unit and are often found in plants and fungi. Triterpenoids from *G. lucidum* have been studied for a range of health benefits. These include potential effects on immune modulation, cardiovascular health, liver protection, and neuroprotection (15–17). Due to their diverse physiological and pharmacological significance, triterpenoids are recognized as the fundamental components responsible to the health benefits of *G. lucidum*. Ganoderic acids and lucidenic acids represent the two primary groups of triterpenoids derived from *G. lucidum* (4, 18). Ganoderic acids are a type of triterpenoid compound that have attracted considerable attention for their potential anti-tumor and anti-inflammatory activities (17). This group of compounds is believed to inhibit the growth of cancer cells by affecting various molecular pathways. There are multiple ganoderic acid derivatives identified in *G. lucidum*, like ganoderic acid A, B, C, D, and so on (17). Lucidenic acids are another type of triterpenoids present in *G. lucidum* that have demonstrated potential health benefits. They are known for their antioxidative, anti-inflammatory, and antitumor activities (19). Lucidenic acids are thought to contribute to the overall health-promoting effects of *G. lucidum*. Lucidenic acids are categorized into several types, including lucidenic acids A, B, C, and others (19). Apart from ganoderic and lucidenic acids, *G. lucidum* contains several other triterpenoids that contribute to its pharmacological properties. Some of these triterpenoids include: ganodermanontriols, ganoderals, ganoderiols, ganodermanondiols, lingzhilactones (4, 5, 20). The collective presence of these triterpenoids contributes to the mushroom’s reputation as a potential health-promoting agent.

Due to the significant role that triterpenoids play, the quantitative content and specific compositional profile of triterpenoids play an important role in determining the overall quality of *G. lucidum* and its associated products. These quantitative and compositional attributes are notably influenced by a range of factors, such as geographical origin, cultivation methodologies, and conditions of harvest, among others. The accurate quantification and identification of triterpenoids across diverse samples of *G. lucidum* may serve as a foundation for the comprehensive assessment of the quality and therapeutic potential of these diverse samples. Therefore, the comprehensive elucidation of triterpenoids in *G. lucidum* samples is of great academic and industrial interest.

The advancement of sophisticated metabolomic methodologies has enabled the comprehensive elucidation of chemical constituents within *G. lucidum* materials. Particularly, untargeted metabolomics approaches that combine cutting-edge LC–MS techniques and chemometric tools offers the advantage of unbiased compound detection and comprehensive coverage of the metabolites (21). This approach allows for extensive chemical profiling and the identification of distinguishing biomarkers that can differentiate various genotypes and phenotypes of *G. lucidum*.

In recent years, there has been growing interest in developing *G. lucidum* strains with targeted enhancements in specific bioactive compounds to maximize their therapeutic potential. Among these, triterpenoids are particularly significant due to their well-documented pharmacological properties, as aforementioned. However, the triterpenoid content in commercially cultivated strains often varies and may be insufficient for maximizing therapeutic effects. This variability is influenced by factors like cultivation conditions and strain genetics, limiting the consistency and potency of bioactive compounds. Recently, we have developed a new *G. lucidum* strain that exhibits a higher concentration of triterpenoids compared to conventional varieties, thereby addressing these limitations. This advancement holds substantial promise for the medicinal mushroom industry, as it could lead to more potent therapeutic products and improved efficacy in clinical applications. The aim of this study is to investigate and compare the metabolite profiles of the conventional strain (GL_V1), which is widely cultivated in Fujian Province, China, and the newly developed enhanced strain (GL_V2), with a particular emphasis on triterpenoid content and composition. By employing ultra-high-performance liquid chromatography coupled with high-resolution mass spectrometry (UPLC-Q-Orbitrap-MS) and advanced chemometric tools, this study seeks to elucidate the potential advantages of the newly developed strain and its implications for the cultivation and therapeutic application of *G. lucidum*.

## 2 Materials and methods

### 2.1 Samples

**Figure 1:**
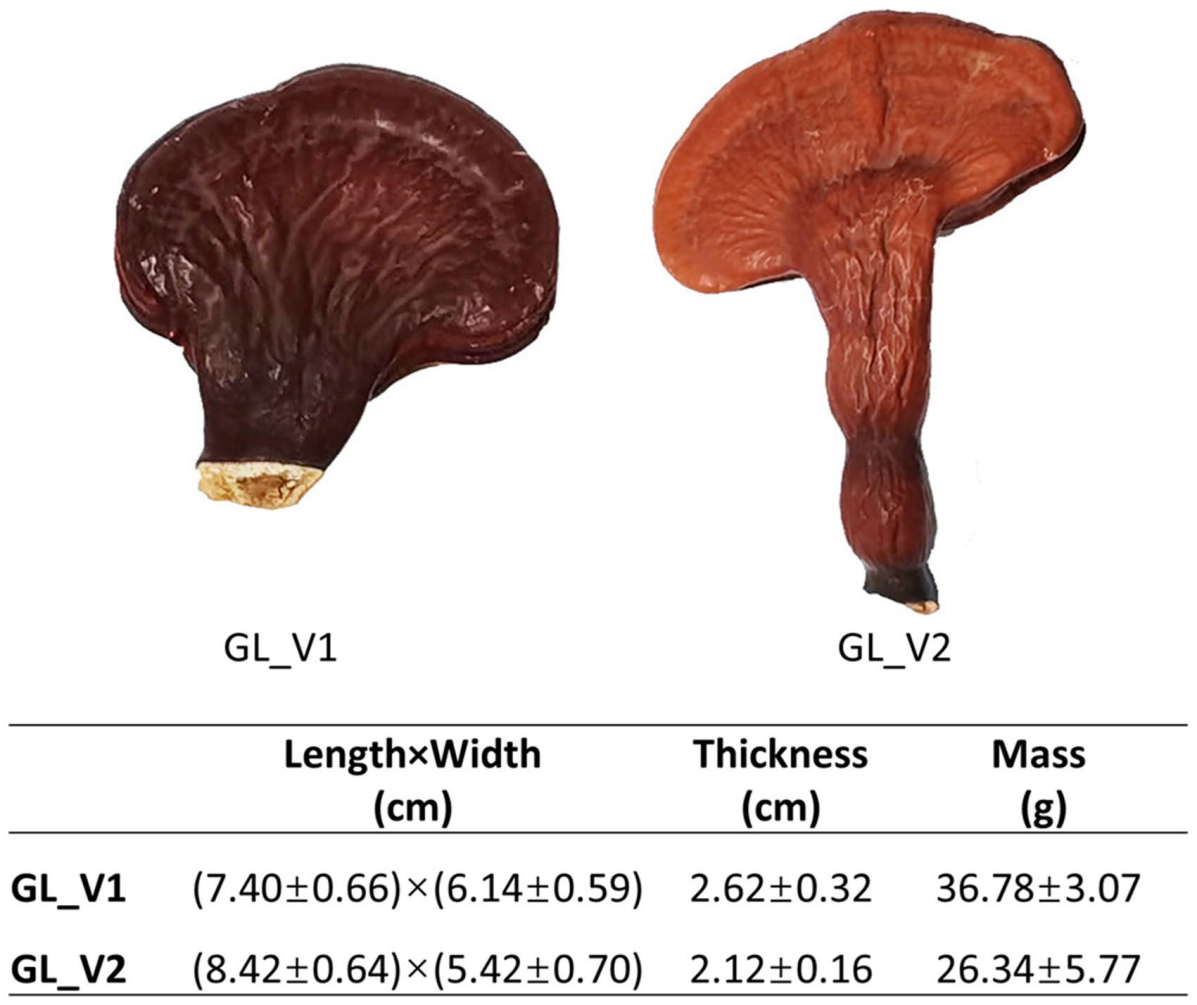
Images of the fruiting bodies of the two *G. lucidum* samples.

The dried fruiting bodies of two *Ganoderma lucidum* varieties, namely GL_V1 and GL_V2, were supplied by GanoHerb Co. Ltd. (Fujian, China). GL_V1 is a widely cultivated domesticated variety of the wild species, while GL_V2 is a newly developed variety obtained through UV-induced mutation breeding technology, featuring increased triterpenoid content. Both varieties were cultured using a substitute cultivation method, as described in our previous study (22). In specific, the growth medium included broadleaf tree sawdust (from oak, chestnut, olive, and peach trees), bran, corn flour, rice malt, and gypsum powder. The cultivation process involved bagging and sterilizing the medium before inoculating it with the fungal cultures and allowing the fruiting bodies to mature. Figure 1 illustrates the fruiting bodies of each variety. For analysis, five individual samples were collected from each variety to assess their chemical composition.

### 2.2 Chemicals

The UPLC grade solvents employed for chromatographic analysis were purchased from CNW Technologies, Inc. (Düsseldorf, Germany). Reagents, including 2-chlorophenylalanine, oleanolic acid, and vanillin were purchased from HC Biotech (Shanghai, China). For LC–MS analysis, 2-chlorophenylalanine served as the internal standard. Ultrapure water was produced using the Millipore Alpha-Q purification system (Millipore, Billerica, MA, United States). Kits for measuring total protein and polysaccharide content were obtained from Jiancheng Bioengineering Institute (Nanjing, China). Other chemicals, of analytical grade, were purchased from Huabo Chemical Reagents Co., Ltd. (Fuzhou, China).

### 2.3 Analysis of total contents of protein, polysaccharides, and triterpenoids

The total protein content in the two types of samples was quantified using the method GB 5009.5–2016 (23), which is the official standard for protein determination in foods. In brief, the dried samples were ground and passed through a 100-mesh sieve. A 100 mg of the resulting powder was placed in a flask with 5.0 mL of 50 mmol/L sodium hydroxide solution, followed by the introduction of 20 mL of biuret reagent. The mixture was vortexed for 15 min and then allowed to settle at room temperature for 30 min. After centrifugation at 4,000 rpm for 5 min, the resulting supernatant was subjected to spectrophotometric analysis at 540 nm for absorbance determination. Protein concentration was calculated by comparing its absorbance value to a calibration curve prepared with bovine serum albumin standards.

The total polysaccharide content in the two types of samples was quantified using the anthrone-sulfuric acid method (24). In brief, 2.0 g of the powdered sample was mixed with 60 mL of water in a flask and left to stand for 1 h. The mixture was then subjected to reflux heating for 4 h. The resulting solution was filtered, and the residue was re-extracted using the same procedure. The combined filtrates were concentrated by rotary evaporation to remove water. The residue was dissolved in 5 mL of water and precipitated with 75 mL of ethanol at 4°C for 12 h. After centrifugation, the precipitate was dissolved in hot water to make a final volume of 50 mL. A 2 mL aliquot of the supernatant was mixed with 6 mL of a sulfuric acid-anthraquinone solution (0.1 g anthraquinone in 100 mL sulfuric acid). After thorough mixing and 15 min of reaction time, the absorbance was measured at 625 nm. Polysaccharide concentration was determined using a glucose calibration curve.

The total triterpenoids content in the two types of samples was quantified using the colorimetric method (25). In brief, 2.0 g of powdered sample was introduced into a flask, and 50 mL of ethanol was subsequently added. Ultrasound (140 W, 42 kHz, 45 min) was used to extract the triterpenoids. After filtering through filter paper, the residue was subjected to a repeat extraction procedure consistent with the initial process. The combined filtrates were merged and centrifugated. For analysis, 0.2 mL of the resulting supernatant was blended with 0.2 mL of vanillin acetic acid solution (0.5 g vanillin in 10 mL acetic acid), in addition to 0.8 mL of perchloric acid. After vigorous shaking, the mixture was heated at 70°C for 15 min. Upon cooling to room temperature, 4 mL of ethyl acetate was added and mixed thoroughly. Absorbance was measured at 546 nm, and triterpenoid concentration was calculated using a calibration curve with oleanolic acid standards. All experiments were performed in triplicate.

### 2.4 Antioxidant activities analysis

#### 2.4.1 Assessment of DPPH radical scavenging activity

The antioxidant capacity was evaluated via DPPH assay following Mishra’s protocol (26). The sample (0.1 g) was mixed with 1 mL 80% methanol, homogenized, and subjected to ultrasonic extraction at 200 W for 30 min. After centrifugation, the supernatant was collected. For the DPPH assay, 150 μL of extract and 150 μL of 0.2 mmol/L DPPH solution were incubated in the dark for 30 min, and absorbance was measured at 517 nm. Controls included methanol and DPPH solution. The scavenging rate was calculated. The experiment was performed in triplicate.

#### 2.4.2 Assessment of hydroxyl radical scavenging activity

Hydroxyl radical scavenging was assessed using a modified Fenton method (27). A mixture of 50 μL sample extract, 50 μL salicylic acid (9 mmol/L), 50 μL FeSO^4^ (9 mmol/L), 50 μL H2O2 (8.8 mmol/L), and 200 μL distilled water was incubated at 37°C for 20 min. Absorbance was measured at 510 nm. Controls replaced sample extract with methanol and H_2_O_2_ with distilled water. Scavenging rates were calculated. The experiment was performed in triplicate.

#### 2.4.3 Assessment of total antioxidant activity

Total antioxidant activity was measured using the FRAP method via a commercially available kit (G0115W, Grace Biotechnology, Suzhou, China). Absorbance was read at 590 nm. A calibration curve of trolox standards (0–20 μmol/mL) was used to calculate the trolox equivalent. The experiment was performed in triplicate.

### 2.5 Untargeted metabolomics analysis

#### 2.5.1 Extraction of samples

The fruiting bodies were pulverized into a fine powder. For each sample, 0.5 g of powder underwent extraction using a mixture of 25% methanol in water, with 2-chlorophenylalanine (1 μg/mL) added as an internal reference. The extraction process involved 60 min of sonication in an ice-water bath. Post-extraction, the mixture was filtered (0.22 μm) and then subjected to centrifugation (12,000 rpm, 20 min, 4°C). A 300 μL aliquot of the resulting supernatant was collected for subsequent metabolomic analysis. To ensure analytical reliability, a quality control (QC) sample was created by pooling equal volumes of supernatant from all individual samples. This QC sample served as a reference for assessing the reproducibility and accuracy of the analytical procedure.

#### 2.5.2 UPLC-Q-Orbitrap-MS analysis

Chromatographic separation of the extracts of all samples was performed on a Vanquish UPLC system (Thermo Fisher Scientific) utilizing an Acquity UPLC BEH C18 column (2.1 × 100 mm, 1.7 μm; Waters). The mobile phase consisted of two solvents: (A) water and (B) acetonitrile, both containing 0.1% formic acid. A flow rate of 0.500 mL/min was maintained. The elution gradient was programmed as follows: 85% A at 0 min, decreasing to 25% A at 11 min, then to 2% A at 12 min. This composition was held until 14 min, after which it returned to 85% A at 14.1 min and was maintained until 16 min. The sample injection volume was set at 5 μL. To ensure analytical reliability, a QC sample was analyzed at the start, midpoint, and end of the sequence, generating triplicate datasets. This approach enabled continuous monitoring of instrumental performance throughout the analytical run.

Following chromatographic separation, a Q Exactive Focus Orbitrap mass spectrometer (Thermo Fisher Scientific) was employed for high-resolution MS analysis in both positive and negative ESI modes. The instrument parameters included a 4.0 kV spray voltage, nitrogen sheath gas at 45 Arb, and auxiliary gas at 15 Arb, with a capillary temperature of 400°C. MS scan range was 100–1,500 m/z, using a full MS resolution of 70,000 FWHM and data-dependent MS/MS resolution of 17,500 FWHM. External calibration was performed prior to the analysis to ensure mass accuracy. Data processing utilized Xcalibur 4.0 software. Raw data was converted to mzXML format using msConvert software and processed with XCMS package in R. Metabolite identification relied on an in-house database (Shanghai Biotree biotech Co.,Ltd.) and public resources like HMDB, METLIN, and M/Zcloud. The final output comprised a matrix of tentative identifications, retention times, and peak areas for detected ion features.

#### 2.5.3 Chemometric analysis

The obtained data matrix was then subjected to chemometric analyses, including Principal Component Analysis (PCA), Hierarchical Cluster Analysis (HCA), Orthogonal Partial Least Squares-Discriminant Analysis (OPLS-DA), through R software and SIMCA-P^+^ 14.0. In these analyses, the peak areas of all variables were subjected to auto-scaling, which served to standardize the differences in their magnitudes and amplitude scales. In order to reduce the number of variables, Variable Importance in Projection (VIP) score of each variable in OPLS-DA was computed. The compounds with VIP scores over 1.0 were selected as the most descriptive compounds for the two different types of samples. PCA was used for initial exploratory analysis, reducing data dimensionality and identifying overall patterns between the two *G. lucidum* strains. HCA was applied to cluster samples based on their metabolomic similarities, allowing for a visual representation of relationships between the groups. OPLS-DA, a supervised method, was employed to enhance discrimination between the two strains by focusing on group differences while minimizing unrelated variations. This approach enabled the identification of key metabolites responsible for the observed differences in triterpenoid profiles.

## 3 Results and discussion

### 3.1 Comparison of total content of protein, polysaccharides, and triterpenoids

Table 1 demonstrates the variations in the levels of protein, polysaccharides, and triterpenoids between the two types of samples. In comparison to GL_V1, GL_V2 demonstrates a 1.54-fold decrease in protein content and a 1.40-fold elevation in triterpenoid content. When examining polysaccharides, the content is similar in both groups of samples. From the perspective of total triterpenoid content, which are recognized as the main bioactive constituents within *G. lucidum*, it is assumed that GL_V2 exhibits superior quality than the commonly grown variety GL_V1.

**Table 1:** The protein, polysaccharide, and triterpenoid contents and antioxidant activities.

	GL_V1	GL_V2
Protein content (mg/g)	155.00 ± 30.99	278.91 ± 4.42 *
Polysaccharide content (mg/g)	8.77 ± 1.72	7.02 ± 1.06
Triterpenoid content (mg/g)	5.89 ± 0.30	8.24 ± 0.08 *
DPPH scavenging rate (%)	31.12 ± 2.39	39.25 ± 3.14 *
Hydroxyl radical scavenging rate (%)	26.72 ± 1.18	29.14 ± 1.16
Total antioxidant activity (μmol Trolox/g)	16.40 ± 0.42	16.58 ± 0.43

Symbol “*” indicates the significant differences between the two types of samples.

### 3.2 Comparison of the antioxidant activities

Antioxidant activities, including DPPH and hydroxyl radicals scavenging activities and total antioxidant capacity, were evaluated in both sample types. As presented in Table 1, the GL_V2 samples demonstrated significantly higher DPPH radical scavenging activity (*p* < 0.05) than that of GL_V1 samples. This enhanced activity in the GL_V2 samples can be attributed to their elevated triterpenoid content.

### 3.3 Validation of the UPLC-Q-Orbitrap-MS method

In order to monitor the reliability of the UPLC-Q-Orbitrap-MS method applied in this study, three QC samples were analyzed during the run of UPLC-Q-Orbitrap-MS. The base-peak chromatograms (BPC) of the triplicate QC samples, acquired under both negative and positive electrospray ionization modes, are presented in Supplementary Figure S1. To further validate the method’s performance, the extracted ion chromatograms (EICs) of the internal standard, 2-chlorophenylalanine, within the QC samples were examined, as shown in Supplementary Figure S2. The EICs were generated for both positive (m/z 200.04728) and negative (m/z 198.03273) modes, with a mass tolerance of less than 5 ppm to ensure high specificity. The uniformity in the BPCs of the QC samples and EICs of the 2-chlorophenylalanine serves as an indicator of the satisfactory stability of the analytical methodology.

### 3.4 Comparison of the general metabolites

**Figure 2:**
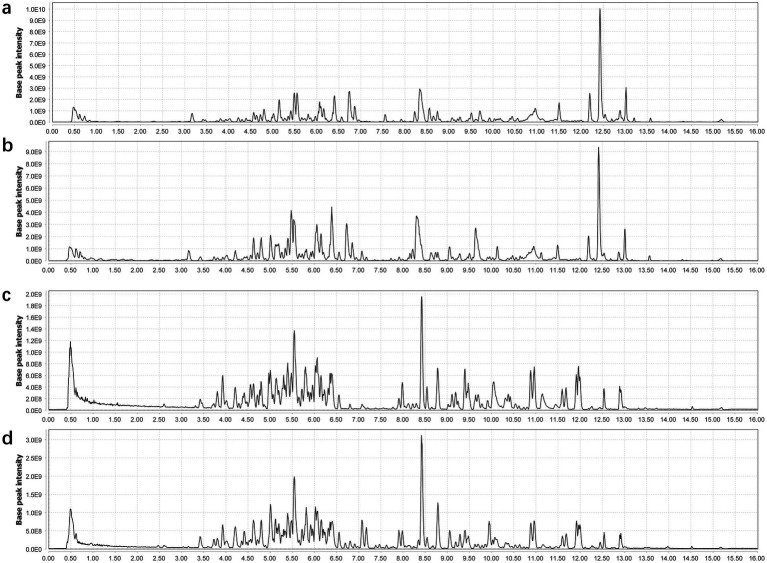
Representative BPC of the fruiting bodies of the two *G. lucidum* varieties acquired in both positive **(a,b)** and negative **(c,d)** ionization modes.

The comprehensive metabolomic analysis of two *G. lucidum* varieties (GL_V1 and GL_V2) was conducted using UPLC-Q-Orbitrap-MS. Figure 2 illustrates the representative BPCs of GL_V1 and GL_V2 samples under both positive and negative ESI modes. It was found that the positive ionization mode demonstrated superior performance, yielding a higher number of peaks with greater intensities compared to the negative mode. When comparing the BPCs between GL_V1 and GL_V2 samples, a similar chemical composition, but with distinctive intensities was revealed. After peak detection, alignment, and filtration, 13,702 and 10,067 ion features were obtained in the two sample types from positive and negative ionization modes, respectively.

**Figure 3:**
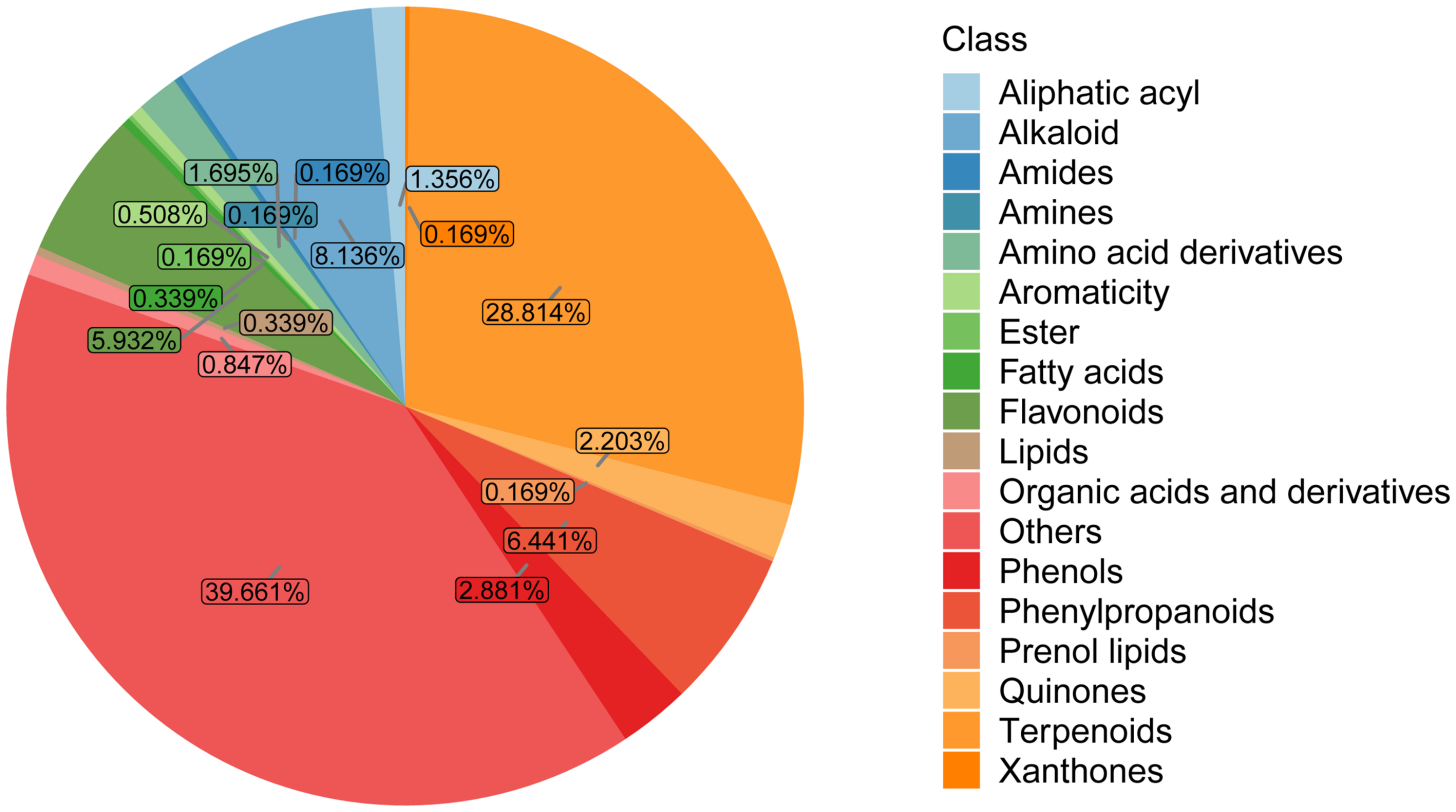
Pie plot of metabolite classification and proportion.

Putative identification of metabolites in GL_V1 and GL_V2 samples was accomplished through the in-house metabolite database and multiple public databases, including the HMDB, METLIN, and *M/Z*cloud. This strategy resulted in the tentative identification of 146 metabolites in negative mode and 443 in positive mode. These metabolites were classified into 18 distinct groups, as demonstrated in Figure 3. Among them, terpenoids emerge as a predominant class, accounting for 28.814% (170 compounds) of all identified metabolites. Other significant classes included alkaloids (8.136%, 48 compounds), phenylpropanoids (6.441%, 38 compounds), and flavonoids (5.932%, 35 compounds), each contributing to the fungus’s complex phytochemical profile. The detailed information of these metabolites is demonstrated in Supplementary Table S1.

Within the terpenoid class, triterpenoids, characterized by their pentacyclic molecular structure, constitute the major constituents. Our analysis identified 86 triterpenoid compounds, including various ganoderic acids, lucidenic acids, ganolucidic acids, ganoderiols, and ganosporelactones. The structural diversity of these compounds underscores the complexity of terpenoid profile in *G. lucidum* fruiting body. Information of the identified triterpenoids can be found in Supplementary Table S2.

When comparing the triterpenoids content between these two types of samples, it was found that GL_V2 exhibits higher triterpenoid content, as evidenced by a 1.77-fold increase in the cumulative peak area of all the triterpenoid compounds identified compared to that of GL_V1. This result was in line with the result that obtained through a colorimetric method. As shown in Table 1, a 1.40-fold increase in the total triterpenoid content within GL_V2 was observed.

### 3.5 General metabolites comparison

**Figure 4:**
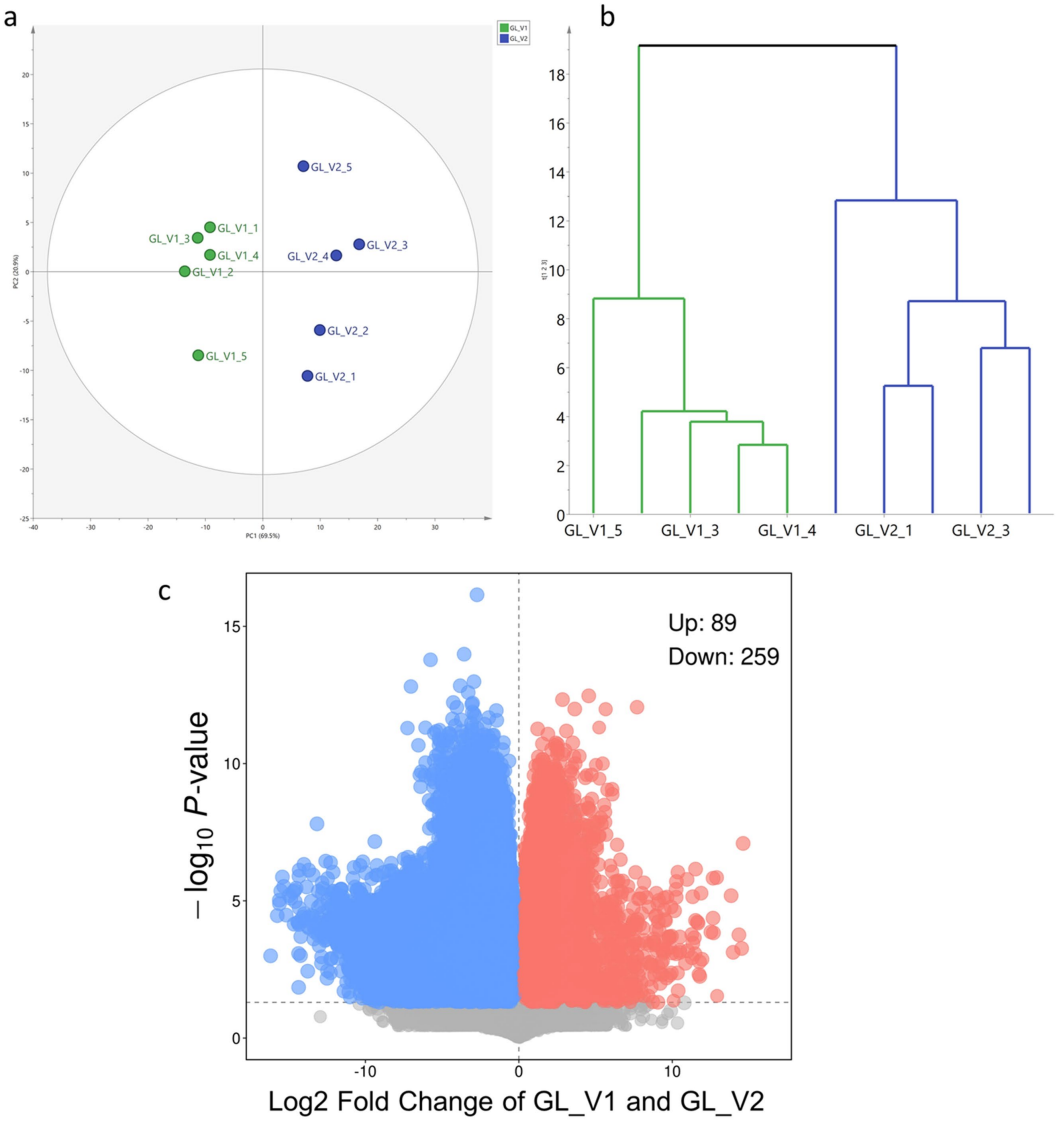
Comparison of general metabolite profiles: PCA score plot **(a)**, HCA dendrogram **(b)**, and volcano plot **(c)**.

To elucidate the general metabolic differences between the fruiting bodies of GL_V1 and GL_V2, multivariate statistical analysis was conducted. All the 589 putatively identified compounds, along with their normalized relative content based on the internal standard, were integrated into a data matrix for the purpose of multivariate statistical analysis. Firstly, PCA, a powerful unsupervised method for dimensionality reduction in complex datasets, was utilized for comparison purposes. The PCA score plot (Figure 4a) revealed a clear segregation of samples into two distinct clusters, corresponding to GL_V1 and GL_V2. The first two principal components (PC1 and PC2) accounted for a substantial 91.1% of the total variance (PC1: 61.3%, PC2: 29.8%). In HCA analysis, the resulting dendrogram (Figure 4b) exhibited a branching pattern similar to the groupings observed in the PCA, further validating the distinct metabolic profile of GL_V1 and GL_V2. The consistency between PCA and HCA results clearly demonstrates the metabolic differentiation between the two *G. lucidum* varieties. Furthermore, we constructed a volcano plot (Figure 4c) to visualize the specific metabolites that contribute most significantly to the differences shown in PCA and HCA. In this plot, each point represents an identified metabolite, with the y-axis representing the -log10 of the *p*-value and the x-axis displaying the log2 of the fold change between GL_V1 and GL_V2 samples. It was found that 89 metabolites were significantly higher in GL_V1 samples compared to GL_V2 samples (*p* < 0.05), and 259 metabolites were significantly lower in GL_V1 samples than in GL_V2 samples (*p* < 0.05). Details of these differentially elevated metabolites can be found in the Supplementary Table S1.

### 3.6 Comparison of triterpenoid profiles

#### 3.6.1 General triterpenoid profile comparison

**Figure 5:**
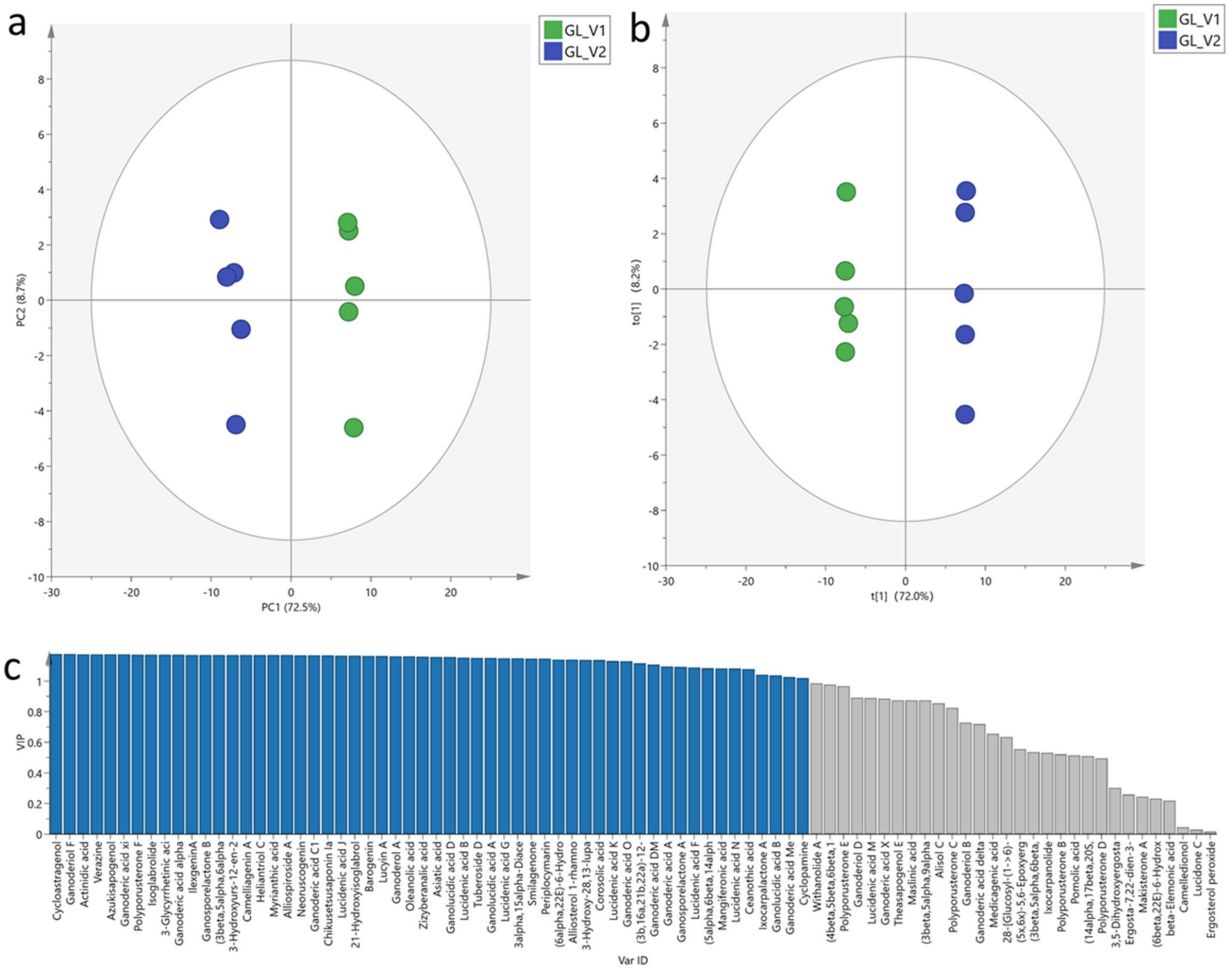
General triterpenoid profiles comparison. PCA score plot **(a)**, OPLS-DA score plot **(b)**, and VIP values **(c)**.

Given the substantial variation in triterpenoid content between GL_V1 and GL_V2 samples and the well-documented therapeutic potential of these compounds, we further conducted a comparative analysis of their triterpenoid profiles. PCA was executed to evaluate the overall distribution of triterpenoid compounds across all samples. The resulting PCA score plot (Figure 5a) revealed a clear segregation of samples into two distinct clusters, corresponding to GL_V1 and GL_V2. The first two principal components (PC1 and PC2) accounted for 97.9% of the total variance (PC1: 83.2%, PC2: 14.7%). Therefore, the results from PCA suggest significant dissimilarity in the general triterpenoid profiles between GL_V1 and GL_V2 samples.

Following PCA, OPLS-DA, which is a supervised discriminant analysis, was performed to explore the distinguishing triterpenoids between GL_V1 and GL_V2 samples. The OPLS-DA score plot (Figure 5b) demonstrated a clear separation between GL_V1 and GL_V2 samples, corroborating the PCA results. The robustness and predictive power of our OPLS-DA model were validated through several statistical parameters. The cumulative R2X, R2Y, and Q2 values were 0.844, 0.999, and 0.996, respectively, indicating excellent model fit and predictive ability. Subsequently, a permutation test (n = 200) was performed to validate the model performance. The resulting intercepts of R^2^ = (0.0, 0.479) and Q^2^ = (0.0, −1.03) indicated the OPLS-DA model was not over-fitting. In order to explore the significantly changed triterpenoids, VIP values for each triterpenoid were calculated, as shown in Figure 5c. Triterpenoids with VIP values greater than 1.0 were considered as the distinguishing metabolites between the two sample types. In total, we identified 56 triterpenoids as such distinguishing metabolites, including cycloastragenol, ganoderiol F, actinidic acid, verazine, azukisapogenol, ganoderic acid xi, polyporusterone F, etc. Detailed information on these 56 differentiating triterpenoids is listed in Supplementary Table S2.

#### 3.6.2 Differentiating triterpenoids

Among the 56 triterpenoids identified as distinguishing metabolites between GL_V1 and GL_V2, we focused our analysis on 20 compounds belonging to well-established bioactive classes in *G. lucidum*: ganoderic acids, ganoderiols, ganolucidic acids, ganosporelactones, and lucidenic acids. These compounds have been consistently detected in various *G. lucidum* tissues, including fruiting bodies, mycelia, and spores. These particular triterpenoid groups are well-documented for their diverse bioactive properties. The detailed information of these 20 differentiating triterpenoid compounds, such as their tentative identification, formula, measured accurate mass, retention time, MS2 fragments, peak areas, fold change values between GL_V1 and GL_V2, and VIP values, is present in Table 2.

**Table 2:** The triterpenoids that were differentially elevated.

ID	Tentative identification	Formula	Measured m/z	RT (min)	Δ m/z (ppm)	MS2	Average peak area		Fold change	VIP
							GL_V1	GL_V2		
1	Ganoderic acid A	C30H44O7	517.31622	4.555	0.42	499.304; 481.297; 463.282; 139.075; 517.309	4.62E+08	1.04E+09	0.44	1.09
2	Ganoderic acid alpha	C32H46O9	575.32003	7.575	1.68	497.289; 92.666; 461.264; 479.28; 69.033	5.00E+05	5.88E+06	0.09	1.17
3	Ganoderic acid C1	C30H42O7	515.29995	7.365	0.10	367.224; 451.283; 497.287; 139.076; 69.033	9.79E+06	1.89E+07	0.52	1.17
4	Ganoderic acid DM	C30H44O4	469.33081	9.448	0.41	469.327; 451.319; 95.085; 393.281; 325.217	1.73E+07	5.95E+07	0.29	1.11
5	Ganoderic acid Me	C34H50O6	555.36467	7.590	0.60	555.366; 61.707; 495.347; 92.664; 139.074	1.48E+03	9.02E+05	0.00	1.03
6	Ganoderic acid O	C30H40O8	529.27954	6.207	0.86	511.271; 139.076; 69.033; 529.276; 483.276	1.30E+07	2.44E+07	0.53	1.13
7	Ganoderic acid xi	C30H42O7	515.29953	5.081	0.90	515.296; 497.287; 69.033; 92.666; 139.076	2.38E+09	6.30E+09	0.38	1.17
8	Ganoderol F	C30H46O3	455.35219	7.184	0.41	455.356; 329.247; 92.664; 81.07; 437.338	2.75E+07	3.82E+08	0.07	1.18
9	Ganoderol A	C30H46O2	439.35696	10.647	0.09	439.359; 421.344; 81.07; 109.101; 69.07	1.85E+07	2.21E+08	0.08	1.16
10	Ganolucidic acid A	C30H44O6	501.31100	3.730	0.95	501.318; 483.306; 121.064; 215.142; 95.085	1.04E+06	4.08E+06	0.25	1.15
11	Ganolucidic acid B	C30H46O6	503.33678	6.462	0.45	503.338; 485.329; 121.065; 139.076; 217.158	1.96E+07	2.27E+07	0.86	1.04
12	Ganolucidic acid D	C30H44O6	501.32079	6.564	0.41	501.321; 483.307; 465.306; 353.245; 139.076	5.90E+07	1.13E+08	0.52	1.16
13	Ganosporelactone A	C30H40O7	513.28388	6.156	0.23	495.275; 513.281; 92.666; 477.265; 365.209	4.42E+09	6.85E+09	0.64	1.09
14	Ganosporelactone B	C30H42O7	515.29958	5.404	0.82	497.288; 479.281; 461.264; 139.076; 115.039	3.40E+09	6.72E+09	0.51	1.17
15	Lucidenic acid B	C27H38O7	475.26900	4.385	0.00	475.263; 439.243; 421.236; 393.244; 457.257	9.10E+08	4.42E+08	2.06	1.15
16	Lucidenic acid F	C27H36O6	457.25885	4.841	0.32	457.303; 439.291; 83.086; 81.07; 71.049	1.21E+08	9.16E+07	1.32	1.09
17	Lucidenic acid G	C27H40O7	477.28518	2.352	0.38	70.065; 423.253; 441.258; 477.283; 293.151	1.38E+07	6.39E+06	2.15	1.15
18	Lucidenic acid J	C27H38O8	491.26446	2.977	0.94	473.253; 491.256; 437.233; 99.044; 419.216	3.80E+07	1.49E+07	2.55	1.17
19	Lucidenic acid K	C27H36O7	473.25359	4.386	0.86	473.298; 455.289; 92.666; 427.242; 81.07	5.25E+07	3.50E+07	1.50	1.13
20	Lucidenic acid N	C27H40O6	461.29003	4.129	0.07	443.275; 425.266; 461.291; 407.256; 121.101	9.74E+07	6.39E+07	1.52	1.08

In this study, we have identified nine different ganoderic acids across all analyzed samples, namely ganoderic acid A, alpha, C1, delta, DM, Me, O, X, and xi. It is noticed that a significant disparity in the abundance of these compounds was observed. Notably, seven of these compounds (ganoderic acid A, alpha, C1, DM, Me, O, and xi) were significantly more abundant in GL_V2 samples (fold change between GL_V1 and GL_V2 < 1.0), as detailed in Table 2. The structural diversity of ganoderic acids arises from variations in their pentacyclic triterpenoid backbone and the presence of different functional groups, including hydroxyls, ketones, and esters. A variety of structurally diverse ganoderic acids, including ganoderic acid A, B, and C, have been identified in *G. lucidum* fruiting bodies, constituting a predominant type of triterpenoids within this fungi (28). The diversity and abundance of ganoderic acids in *G. lucidum* can vary depending on the specific *G. lucidum* variety and cultivation techniques employed. From a functional perspective, ganoderic acids are well-known for their pharmacological properties, such as anti-oxidant, anti-inflammatory, anti-cancer, and immunoenhancement effects (17). Therefore, the higher abundance of these compounds in the GL_V2 samples suggests that products derived from GL_V2 may offer enhanced therapeutic potential compared to those derived from GL_V1. This finding holds particular significance for the pharmaceutical industry, as it indicates the possibility of optimizing the production of *G. lucidum*-based health products by selecting and cultivating specific varieties.

Four different ganoderols, including ganoderol A, B, D, and F, were detected across all analyzed samples. Utilizing the OPLS-DA analysis, it was found that ganoderol A and F emerged as the key distinguishing triterpenoids between the two sample types, as indicated by their VIP values exceeding 1.0. This finding suggests that the relative abundance of ganoderol A and F can serve as a marker to differentiate between GL_V1 and GL_V2 samples. Ganoderols are a notable group of triterpenoids abundantly found in *G. lucidum*. These compounds are distinguished by their complex tetracyclic triterpenoid core structure, featuring multiple interconnected rings and diverse functional groups. The ganoderol group includes various types, such as ganoderol A, B, C, D, and F (29). Among the ganoderol compounds, ganoderol A stands out as a prominent constituent within *G. lucidum*. Ganoderol A falls under the category of lanostane-type triterpenoids, featuring a tetracyclic structure composed of four fused rings. Its noteworthy attributes have garnered substantial research attention, particularly due to its potential health-enhancing properties, such as anti-oxidative and anti-cancer activities (30). Our findings unveiled considerable disparities in the levels of ganoderol A and ganoderol F between the two sample varieties, with GL_V2 samples exhibiting a remarkable 11.9-fold increase in ganoderol A content and 13.9-fold increase in ganoderol F content in comparison to GL_V1 samples.

Three ganoderic acids, including ganoderic acid A, B and D, were identified across all analyzed samples. Ganoderic acids belong to the category of lanostane-type triterpenoids and are recognized constituents of *G. lucidum* (31). Several forms of ganolucidic acids, such as ganolucidic acid A, B, C, D and E, have been documented in the literature (6). In this study, it was found that all the three detected ganoderic acids were in higher abundance in GL_V2 samples.

Ganosporelactone A and ganosporelactone B are initially isolated from *G. lucidum* spores. Biosynthetically, these two triterpenoid lactones are thought to originate from the lanostane skeleton through an intramolecular cyclization involving C16 and C23, resulting in a distinctive lactone moiety (32). Ganosporelactones are renowned for their remarkable health promoting effects, such as anti-oxidantive, anti-inflammatory, and anti-cancer properties (33). As shown in Table 2, the GL_V2 samples showed a 1.55-fold increase in ganosporelactone A and a 1.97-fold increase in ganosporelactone B when comparing those in the GL_V1 samples. Higher concentration of these compounds in the GL_V2 samples suggests their higher therapeutic potentials.

Additionally, we identified seven distinct lucidenic acids, including types B, F, G, J, K, M, and N. Interestingly, all except lucidenic acid M were found in higher abundance in GL_V1 samples. This pattern contrasts with the distribution observed for other triterpenoid compounds such as ganoderic acids, ganoderiols, ganolucidic acids, and ganosporelactones. Lucidenic acids, characterized by their distinct C27 lanostane skeleton and a carboxyl group in the side chain, constitute the second most abundant group of triterpenoids in *G. lucidum*, after ganoderic acids (19). To date, researchers have identified 22 different lucidenic acids in this fungus. A comprehensive review by Zheng and colleagues recently explored the origins, quantities, molecular structures, and biological activities of these compounds (19). Our findings suggests that the GL_V1 variety may possess a distinct composition of lucidenic acids, potentially offering unique properties or benefits.

Taken together, when considering the 20 commonly identified triterpenoids in *G. lucidum* with VIP values higher than 1.0, it is notable that only six lucidenic acids displayed higher concentrations in the GL_V1 samples. Conversely, the remaining triterpenoids, comprising seven ganoderic acids, two ganoderiols, three ganolucidic acids, and two ganosporelactones, exhibited elevated levels in the GL_V2 samples. This observation was in line with the finding that GL_V2 samples have higher total triterpenoid content. The increased triterpenoid content in GL_V2 is strongly correlated with its enhanced antioxidant activity. Specifically, the higher levels of ganoderic acids, which are known for their potent antioxidant properties, likely contribute significantly to the observed increase in antioxidant activity. These compounds, by scavenging free radicals and inhibiting lipid peroxidation, help protect cells from oxidative damage. In addition, ganoderiols and ganolucidic acids, which were also found to be more abundant in GL_V2, have shown similar antioxidant potential in previous studies. The synergistic effects of these triterpenoids may explain the superior antioxidant activity of GL_V2, making it a promising candidate for therapeutic applications focused on oxidative stress-related diseases.

While this study provides valuable insights into the triterpenoid profiles of GL_V1 and GL_V2, it is important to note that *G. lucidum* contains a wide range of bioactive compounds, such as polysaccharides, which were not addressed in this analysis. Polysaccharides have been shown to contribute significantly to the therapeutic properties of *G. lucidum*, particularly in immune modulation and anti-inflammatory activities. Therefore, the exclusion of these compounds represents a limitation of the current study. Future research could build on these findings by exploring the full spectrum of bioactive compounds in GL_V2, which would provide a more comprehensive understanding of its therapeutic potential. Additionally, future work could investigate the molecular and ecological factors shaping triterpenoid biosynthesis in *G. lucidum*. Exploring the underlying mechanisms of mutation breeding and its impact on metabolic pathways could provide deeper insights into the potential for further enhancing the medicinal properties of this fungus.

## 4 Conclusion

This study utilized an untargeted metabolomic approach based on UPLC-Q-Orbitrap-MS to investigate the variations in triterpenoid composition in the fruiting bodies of two selected *G. lucidum* varieties. The newly developed strain, GL_V2, demonstrated a significantly higher concentration of triterpenoids and DPPH radical scavenging activity compared to the widely cultivated conventional strain, GL_V1. The advanced UPLC-Q-Orbitrap-MS-based metabolomic approach allowed for a detailed elucidation of the triterpenoid diversity, identifying a range of ganoderic acids, lucidenic acids, ganoderiols, and other key triterpenoids with known pharmacological activities. Statistical analyses, including PCA, HCA, and OPLS-DA, confirmed the distinct metabolomic and triterpenoid profiles of the two varieties, highlighting GL_V2’s superior triterpenoid content and structural diversity. The results suggest that GL_V2 holds substantial promise for the medicinal mushroom industry, as its enhanced triterpenoid content may offer greater therapeutic potential. This strain could lead to the development of more potent *G. lucidum*-derived products. Moreover, the findings of this study provide a valuable foundation for further research into the genetic, ecological, and biochemical factors that contribute to triterpenoid biosynthesis in *G. lucidum*. Additionally, the enhanced triterpenoid content and antioxidant activity of GL_V2 highlight its potential for the development of more effective nutraceutical and pharmaceutical products, offering new opportunities for the production of high-quality *G. lucidum*-based health supplements and therapeutic formulations.

## Data Availability

The original contributions presented in the study are included in the article/Supplementary material, further inquiries can be directed to the corresponding author/s.
