# Supplementary material for: Unveiling triterpenoid superiority in a newly developed Ganoderma lucidum variety through untargeted metabolomics approach
Source: Front Nutr. 2025 Apr 10;12:1541162. doi: 10.3389/fnut.2025.1541162 (PMC12018227; doi:10.3389/fnut.2025.1541162)
Supplement: Supplementary file 1 [file Data_Sheet_1.zip › Triterpenoid Profiles in GL_03 SI/SI_Figure S1&S2.docx]

Figure S1.


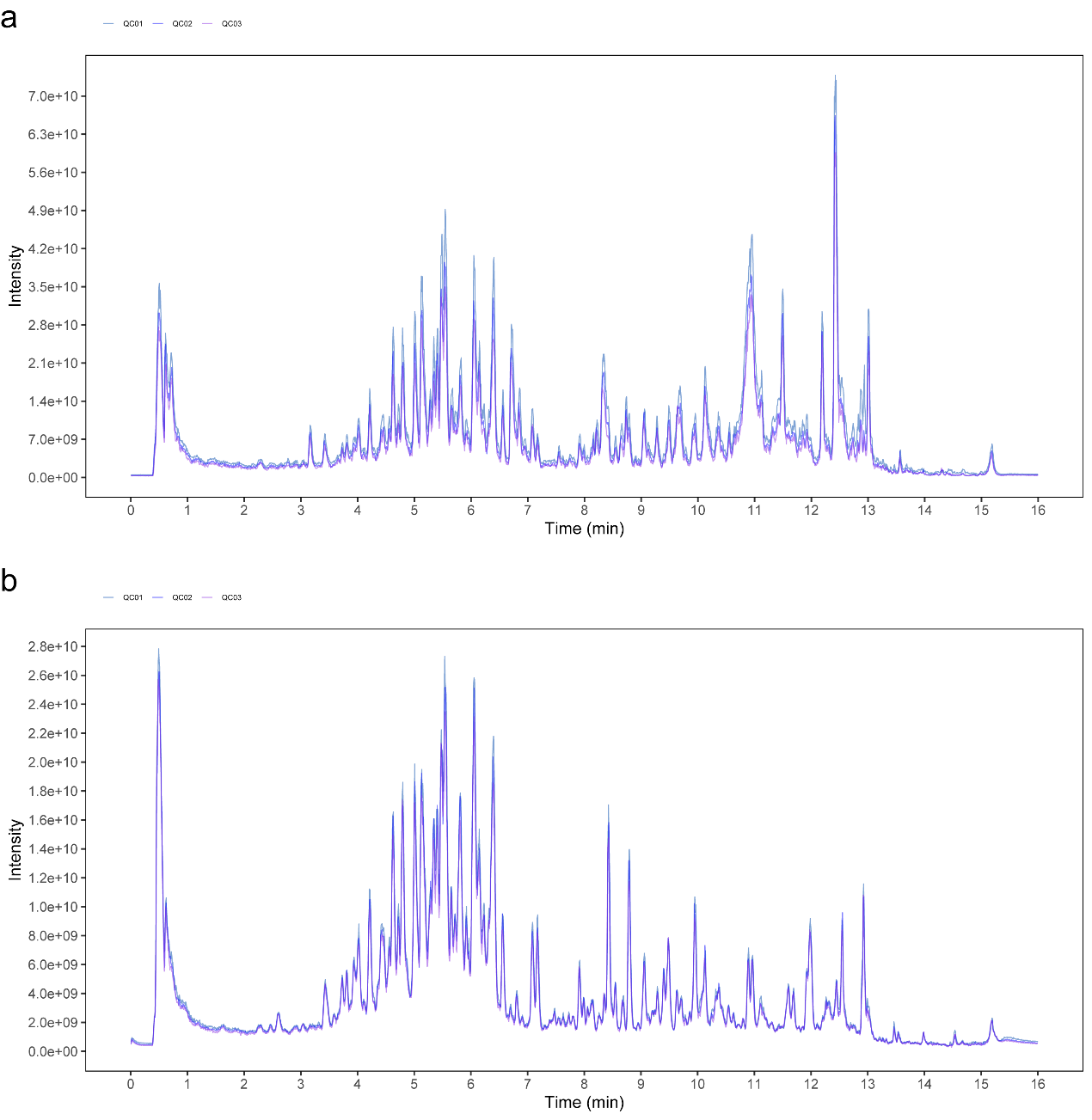


**Figure S1.** Base peak chromatograms of QC sample in positive (A) and negative (B) ionization modes

Figure S2


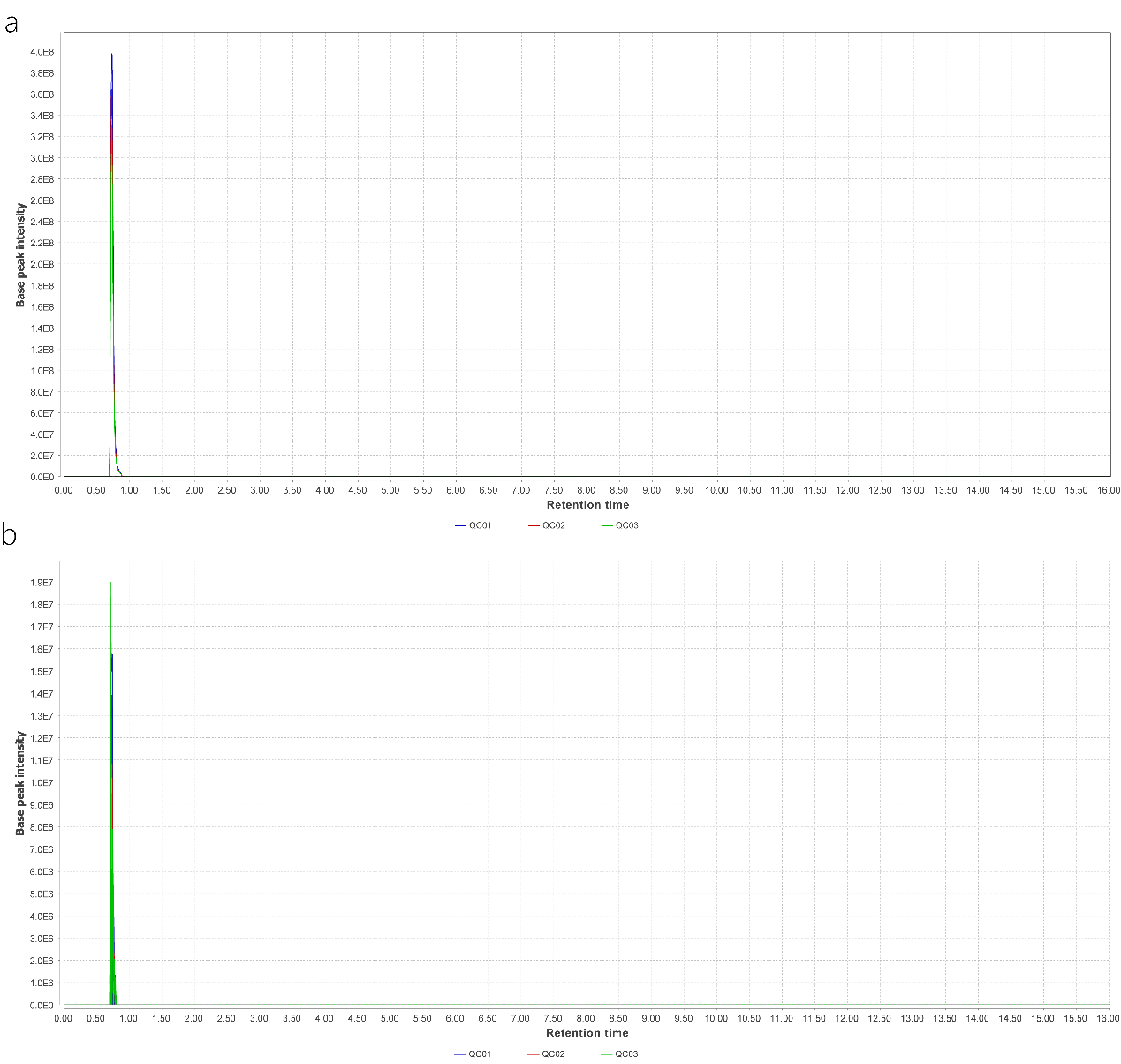


**Figure S2.** The extracted ion chromatograms of the internal standard 2-chlorophenylalanine within the QC samples.
